# Comment on Rosell-Cardona et al. Dietary Spray-Dried Porcine Plasma Reduces Neuropathological Alzheimer’s Disease Hallmarks in SAMP8 Mice. *Nutrients* 2021, *13*, 2369

**DOI:** 10.3390/nu13114053

**Published:** 2021-11-12

**Authors:** Artemissia-Phoebe Nifli

**Affiliations:** 1 Faculty of Medicine, University of Thessaly, Viopolis, 415 00 Larissa, Greece; nifli@med.uth.gr or f_nifli@hotmail.com; 2 Faculty of Nursing, University of Thessaly, Gaeopolis, 411 10 Larissa, Greece; 3 Department of Agriculture Crop Production and Rural Environment, University of Thessaly, 384 46 Nea Ionia, Greece; 4 Larissa Association of Alzheimer’s Disease and Related Disorders (ΕΕΝAΛ), Chatzigianni 3, 412 21 Larissa, Greece

Interventions focusing on dementia risk and/or dementia modification in association with senescence are essential, given the unfavourable demographics [1,2,3].

Single micronutrient or fatty acid supplementation has failed to provide strong scientific evidence. A combination (Fortasyn™Connect, Souvenaid^®^) produced conflicting results in either prodromal Alzheimer’s disease (AD) or mild AD dementia well designed randomized controlled trials [4]. Thus, repurposing other neutraceuticals could be advantageous, as recently reported [5].

The introduction of animal blood in animal feed had been investigated before 1929. Major safety/quality issues have been resolved, justifying its use partly or *in toto* [6]. For humans, animal blood consumption arose as a vastly controversial, traditional sustainability practice worldwide.

In antiquity, blood was the main ingredient of the “black broth”, an everyday preparation relished by Spartan elders sparing the meat, but disgusting to other Mediterraneans [7]. Nowadays, Finland, France, Norway, Poland, Portugal and Sweden survey explicitly households about blood or blood/black food items (pudding/sausages, bread, pancakes) [8], and Bury, Greater Manchester, England, UK has been reported as the “world black pudding capital” [9]! Sweden also allows for black pudding provisions in school lunches once monthly. So, why are we lacking specifics?

Blood containing food items are often grouped under canned meat and meat products, offal, meat dishes or soy bean products (i.e., blood tofu). Some are considered delicacies, thus less accessible, and consumption scarce; European Union has certified Protected Geographical Indication to 23 products [10]. Most important, (raw) blood consumption is usually part of a restricted diet, indicative of food insecurity [7,11,12].

It is possible though that these choices promote survival and health. A raw blood diet in early weaned Maasai babies [11], contributing iron and protein and strengthening the gut barrier, would be a better alternative to the breast milk of a poorly fed mother. Nutrient digestibility and bioavailability may be an issue as well. Inuit have persistently requested access to “country food”, especially in health care settings. In Nunavut, hospitals were the first to offer raw bloody meat, fish, skin or offal, and this diet has been considered superior to *i.v.* or parenteral solutions.

Comparing the biological activity among fresh, processed and isolated blood products, and identifying components of particular interest will be challenging. Major plasma proteins are also notorious for their interactions with micronutrients [13], thus probable not to act alone. Furthermore, the limited epidemiological data do not support superior cognitive scores in regions where regular blood consumers reside [1,2,3], while the emergence of African Swine Fever Virus and the amyloidogenicity of prion proteins of resistant species raise severe concerns about potential applications. Regardless, the work of Rosell-Cardona et al. [5] provoked us to revisit an old nutrition landscape with fresh, yet judicious eyes.

## Data Availability

Not applicable.
